# Change in the Neural Response to Auditory Deviance Following Cognitive Therapy for Hallucinations in Patients With Schizophrenia

**DOI:** 10.3389/fpsyt.2020.00555

**Published:** 2020-06-12

**Authors:** Verner Knott, Nicola Wright, Dhrasti Shah, Ashley Baddeley, Hayley Bowers, Sara de la Salle, Alain Labelle

**Affiliations:** ^1^School of Psychology, University of Ottawa, Ottawa, ON, Canada; ^2^Clinical Neuroelectrophysiology and Cognitive Research Laboratory, University of Ottawa Institute of Mental Health Research, Ottawa, ON, Canada; ^3^Department of Psychiatry, University of Ottawa, Ottawa, ON, Canada; ^4^Schizophrenia Program, The Royal Ottawa Mental Health Centre, Ottawa, ON, Canada; ^5^Department of Psychology, University of Guelph, Guelph, ON, Canada

**Keywords:** schizophrenia, auditory hallucinations, cognitive behavioral therapy, auditory cortex, auditory sensory processing, mismatch negativity

## Abstract

Adjunctive psychotherapeutic approaches recommended for patients with schizophrenia (SZ) who are fully or partially resistant to pharmacotherapy have rarely utilized biomarkers to enhance the understanding of treatment-effective mechanisms. As SZ patients with persistent auditory verbal hallucinations (AVH) frequently evidence reduced neural responsiveness to external auditory stimulation, which may impact cognitive and functional outcomes, this study examined the effects of cognitive behavioral therapy for voices (CBTv) on clinical and AVH symptoms and the sensory processing of auditory deviants as measured with the electroencephalographically derived mismatch negativity (MMN) response. Twenty-four patients with SZ and AVH were randomly assigned to group CBTv treatment or a treatment as usual (TAU) condition. Patients in the group CBTv condition received treatment for 5 months while the matched control patients received TAU for the same period, followed by 5 months of group CBTv. Assessments were conducted at baseline and at the end of treatment. Although not showing consistent changes in the frequency of AVHs, CBTv (vs. TAU) improved patients' appraisal (p = 0.001) of and behavioral/emotional responses to AVHs, and increased both MMN generation (p = 0.001) and auditory cortex current density (p = 0.002) in response to tone pitch deviants. Improvements in AVH symptoms were correlated with change in pitch deviant MMN and current density in left primary auditory cortex. These findings of improved auditory information processing and symptom-response attributable to CBTv suggest potential clinical and functional benefits of psychotherapeutical approaches for patients with persistent AVHs.

## Introduction

Auditory verbal hallucinations (AVHs), defined as perceptions or subjective experiences of “hearing voices” without corresponding external auditory stimulation, occur with a high frequency of up to 60% to 80% in patients with schizophrenia (SZ) (1). Reflecting a diverse phenomenological experience, AVHs can involve words, sentences, or conversations (with varied clarity, loudness, and spatial locations) spoken as commands, comments, insults, or encouragements by familiar or unfamiliar single and/or multiple voices (in first, second, or third person) (2).

Although the causes of AVHs are still unclear, improved understanding of the neural basis of AVHs has been forthcoming from functional magnetic resonance imaging (fMRI) studies which have shown elevated activation of brain regions associated with auditory stimulus processing, speech generation, and speech perception during the experience of active hallucinations (vs. silent rest) (3–6). Paradoxically, although in sensory cortices hyper-excitable neuronal states are typically associated with enhanced exogenous induced processes (7–10), AVHs have been associated with reduced neuronal activation of the auditory cortex in response to external auditory stimulation (11). These opposing findings in hallucinating patients of increased activation of the auditory cortex in the absence of external stimulation and reduced activation of the auditory cortex in response to externally presented speech and non-speech sounds have been interpreted as evidence for competition between internally generated and externally originating neural activity in the auditory cortex for the attentional resources of the hallucinating patient (11). Also evidenced in SZ patients who are prone to AVHs (vs. patients who have never hallucinated), diminished neural responsiveness to external auditory stimulation is believed to affect the functional cost of an auditory cortex that is thought to be tonically **“**tuned on” and “tuned in” to the internal channels broadcasting hallucinating stimuli, with the preferential endogenous processing of AVHs resulting in the “saturation” of neuronal resources and resulting in limited capacity for the exogenous processing of external auditory stimuli (12, 13).

Further evidence that the auditory cortex in hallucinating patients is overly sensitive to activation arising from internal processing, while being less responsive to external stimulation, comes from electrophysiological studies assessing cortical responsiveness to auditory stimuli with electroencephalographically (EEG)-derived event-related potential (ERP) components that have been shown to be generated in the auditory cortex and have been extensively used to document profound early auditory information processing (EAIP) deficits in SZ (14). Patients with SZ have been found to be impaired with respect to two aspects of EAIP: inhibiting intrinsic responses to redundant stimuli (to prevent sensory overload), and facilitating/detecting potentially salient stimuli (for extended higher-order processing and response) (15). These elementary pre-attentive auditory input deficits in SZ are reflected in two candidate ERP endophenotypes, one of which includes P50 sensory gating as a measure of inhibitory failure. This inhibitory deficit is indexed in SZ both by minimal suppression of P50 (an early central-maximum positive scalp component elicited at ~50 ms in response to the second stimulus [S_2_] of click pairs [S_1_-S_2_]), and by a diminished S_1_ P50 amplitude (16). A second ERP endophenotype of EAIP dysfunction in SZ, mismatch negativity (MMN), is a frontal maximum negative scalp component at ~150 to 200 ms which indexes automatic acoustic deviance detection and, in SZ, exhibits a reduced amplitude in response to changes in physical or abstract features in auditory oddball paradigms (17).

Although both of these ERP-indexed elementary sensory processes (auditory gating and auditory charge detection) have been consistently shown to be abnormal in SZ, our findings indicated a significant worsening of these brain sensory functions in patients who hallucinate (trait positive) as: (1) increasing negative affective content of AVHs was inversely related with S_1_ P50 amplitude (18); (2) SZ hallucinators (vs. non-hallucinators) exhibited smaller MMNs to changes in pure tone stimuli (19), with MMN reduction being more evident with increasing trait ratings of hallucinatory activity (20); and (3) SZ hallucinators (vs. healthy controls) showed smaller MMNs to pure tone and speech deviant stimuli (21). Furthermore, in SZ patients who are prone to hallucinate, we observed diminished involuntary attentional orienting to speech stimuli (evidenced by a reduction in a later [~300 ms] frontocentral positive [P3_a_] scalp component), suggesting an impairment in the ability of human speech deviations to capture attention (22). Together with findings of reduced amplitude of the N1 component of the auditory ERP during hallucinating states (23), observed ERP deficits in sensory registration (N1), sensory inhibition (P50), sensory discrimination (MMN), and stimulus selection (P3_a_) within the auditory modality are consistent with the “saturation” hypothesis of AVHs. The resulting competitive outcome favoring resource allocation to the processing of internal auditory signals may in part explain the profound behavioral performance deficits of SZ patients during auditory discrimination tasks (24).

AVHs are associated with high levels of distress likely related to idiosyncratic beliefs or cognitive appraisals involving control, power, voice identity, authority, and consequences of not complying with the voices (25–27). Despite adequate dosages of antipsychotic drugs, AVHs are drug resistant in ~25% of SZ patients, and become chronic, causing an impaired quality of life (28) and diminished cognitive capacity, with the latter playing a key role in functional outcome (29). Cognitive behavioral therapy (CBT) has been suggested as a complement to pharmacotherapy for targeting psychosis in treatment resistant cases (30–32). Reviews (33–37) and multiple meta-analyses (38, 39, 40–42) on the effectiveness of specialized cognitive behavioral therapy for psychosis (CBTp), developed, and recommended as an adjunctive treatment for decreasing distress in patients with persistent AVHs (43, 44), found a modest but significant positive impact on positive symptoms, negative symptoms, and general psychopathology. The proposed mechanism of change resulting from CBTv is through changes in beliefs about voices as well as enhancing coping skills (33).

In contrast to CBTp, which is aimed at a broad array of symptoms, administering tailored therapies for specific symptoms and using a recommended symptoms specific approach such as CBT for voices (CBTv) (34, 40) has shown effectiveness in individual and group sessions. In three randomized controlled group CBTv trials, improvements have been found not only in positive and general symptoms, but also in self-esteem, effective coping strategies and social functioning, as well as reductions in voice frequency and perceived voice power (35, 45–49).

### The Present Study

The ultimate goal of CBTv is to help patients cope with auditory hallucinations, which would presumably translate into improved external auditory information processing, and to improved functioning. The primary aim of this pilot study was to examine change in the neuronal response to auditory stimulation following an integrated group CBTv trial which would incorporate the use of both acceptance and commitment therapy (ACT) to modify painful and stressful thoughts and emotions arising from voices (50–52), and attentional training (ATT) to reduce the attentional capture by emotionally salient voices (53, 54). Effective cognitive strategies that are able to reduce AVH saturation of sound perception neurocircuitry may free up resources for external auditory processing in limited capacity auditory cortical networks. At the sensory processing level, the MMN may be an ideal probe for indexing treatment associated with functional changes in the auditory cortex as: it can be rapidly assessed and it is highly stable over time (test-retest ranging from 0.60 to 0.80) (55, 56); it is an automatic sensory process that is relatively free of attentional and motivational confounds that influence effort-demanding, higher order cognitive operations (57); and finally, because MMN has strong external face validity (in that it is positively related to performance in behavioral tasks of sound discrimination) (58, 59), and its impairment in SZ is positively correlated with cognitive (memory) (60) and executive functioning deficits (61, 62), social skills acquisition (63), and global daily functioning (64–67).

In addition to our study's primary objective of using MMN to index CBTv-induced changes in neural correlates of auditory discrimination of pure tone deviants, a complimentary objective was to conduct a regions of interest (ROI) analysis on deviance-elicited source localized activity in bilateral primary (pAC) and secondary auditory cortices (sAC), putative regions implicated in AVHs, and the main cortical areas of MMN generation (68–76). Hypothetically, although we do not necessarily expect changes in AVH topography (i.e., frequency and quality of voices), within the “saturation” model we generally predicted that CBTv, in reducing resource-demanding processing of internal (voices) stimulation, will allow for increased processing of external auditory stimulation. At the neural level, we specifically hypothesized that increased exogenous processing following CBTv will be evidenced by greater MMN responses to auditory deviants, and by greater deviant-elicited activation in the primary auditory cortex (pAC), and specifically the left pAC as this is the main brain region in SZ hallucinators that exhibits both increased activation in the absence of an external stimulus and decreased activation in the presence of an external auditory stimulus (11). CBTv-induced changes in symptoms were expected to correlate with changes in deviance elicited MMN and auditory cortex responses. MMN changes with CBTv were also predicted to be related to response changes in the auditory cortex.

## Methods

### Study Participants

The study was approved by the Research Ethics Board of the Royal Ottawa Mental Health Centre and the University of Ottawa. The study recruited twenty-five (10 women, 15 men) individuals with schizophrenia (SZ: M=45.95 years, SD=12.60) from the Outpatient Schizophrenia Program of the Royal Ottawa Mental Health Centre, all of which were diagnosed by trained psychiatrists using the Structured Clinical Interview DSM-IV-TR (SCID-I) (77). Patients included in the study: (i) were between the ages of 18 and 60 years; (ii) reported a consistent history of auditory verbal hallucinations over the course of their illness; (iii) exhibited a score of 3 or greater (reflecting mild or greater auditory/verbal hallucinatory experience) on the hallucination item of the Positive and Negative Syndrome Scale (PANSS) (78), and a score less than 65 on the total PANSS score (to screen out individuals with severe level of symptoms and severe impaired functioning that would impact their ability to participate in group CBTv); (iv) reported no history of neurological conditions or head injury; (v) were clinically stable, as indicated by no significant changes in symptoms or medication, for at least the 3-month period prior to testing; (vi) were being treated only with one of the atypical antipsychotics as their primary medication; (vii) were willing to participate in 5 to 6 months of CBTv in addition to their usual treatment; and (viii) displayed normal hearing (threshold < 30 dB SPL) as assessed by audiometric testing.

### Treatment Design

**Figure 1:**
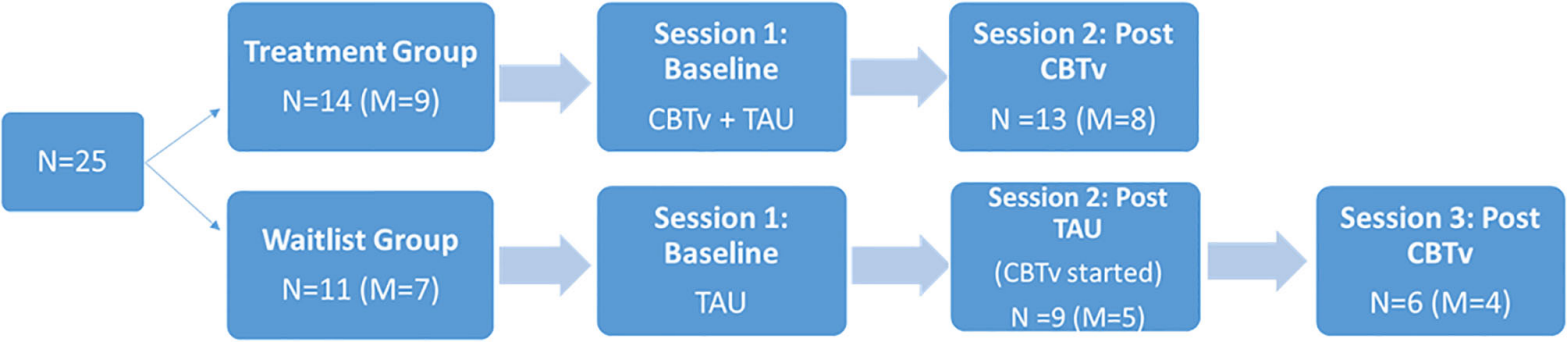
Flow chart of treatment design.

Following a parallel group design, 14 (8 males) of the 25 patients were randomly assigned to receive CBTv for 5 months in addition to their usual treatment (CBTv group) and eleven (7 males) were randomly assigned to continue their treatment as usual (TAU group). The recruitment and creation of groups involved: (i) a patient referral through hospital psychiatrist to the study team; (ii) the introduction of the study requirements and involvement by the study team and consent from participants; (iii) completion of screening session to ensure patients met the study requirements; and (iv) random assignment to treatment groups. In the CBTv group, patients received CBTv for 5 to 6 months, while patients in the TAU were followed for the same time period. Following completion, TAU patients then completed 5 months of CBTv treatment (**Figure 1**). The two laboratory test sessions, one at baseline and one at follow-up, included electrophysiological recordings, assessment of psychiatric symptoms, and completion of questionnaires relating to AVHs.

Of the fourteen patients assigned to the CBTv condition, thirteen patients (8 males) completed all assessments at baseline and follow-up and provided usable EEG data. Of the eleven patients recruited in TAU group, nine (5 males) completed all assessments at baseline and follow-up and provided usable EEG data. Of the nine patients who completed the TAU group, six completed all assessment at baseline, follow-up, and post-CBTv, and provided usable EEG data. All patients who completed the study continued with their regular medication and psychosocial interventions throughout the study period. The main reasons for attrition or exclusions from the study were: (i) consent withdrawal; (ii) incomplete or unusable EEG data at one or both time points (e.g. noisy EEG data, less than 40 clean EEG epochs per deviant stimulus, and missing EEG channels; (iv) medication change; and (v) onset of medical illness.

### CBTv Protocol

Consistent with the NICE (77) and PORT (78) guidelines, group CBTv was delivered using a manualized approach, where prescribed goals and techniques to be used during treatment sessions are outlined and followed throughout treatment. The treatment was implemented by one expert CBTv therapist (N.W.), following a session-by-session treatment manual. Conducted in eighteen planned sessions over 5 months, and facilitated by highly trained group leaders, the CBTv intervention incorporated CBT strategies for positive symptoms, and ATT as well as ACT within a CBT framework. The 18 session group CBTv was administered on a weekly basis for 5 months (during the last 2 months, sessions were spread out to every two weeks). Each CBTv group had approximately nine participants and each participant had a copy of the participant manual, which included all homework/practice assignments. Adherence to the CBTv protocol across the groups was assessed by adherence to the treatment manual and measured by the Cognitive Therapy Scale for Psychosis (CTS-Psy) (79).

### Symptom Assessment

Patients in the CBTv group were assessed independently at two test sessions: at baseline, and at follow-up at the end of CBTv (5 months after baseline). The TAU group was assessed at three test sessions: baseline, at follow-up at the end of waitlist period (5 months after baseline) and at the end of CBTv (10 months after baseline). The following clinical outcome measures were implemented:

*Positive and Negative Syndrome* Scale (78). The Structured Clinical Interview for the PANSS is a 30-item rating scale designed to measure the presence and severity of psychopathology in patients with SZ, schizoaffective disorder, and other psychological disorders. The PANSS was completed by a trained clinician following a semi-structured interview format and using available clinical information. The clinician was blind to the group assignments. Each item was rated by the clinician on a Likert scale ranging from 1 (not present) to 7 (extremely severe). Three subscales scored were derived: Positive Symptoms scores (possible range of scored: 9–49); Negative Symptoms Scores (possible range of scores: 7–49); and General Symptoms Scores (possible range of scores: 16–112).

*The Psychotic Symptom Rating Scales (PSYRATS)* (80). The PSYRATS includes two scales designed to measure the severity of a number of dimensions of auditory hallucinations and delusions. Only the Auditory Hallucinations subscale was administered to the patients, which includes an 11-item scale that assesses dimensions of auditory hallucinations. The items include frequency, duration, location, loudness, amount and intensity of distress, amount and intensity of negative content, disruption, controllability, and number of voices. Symptoms scores are rated on a 5-point ordinal scale (0–4). Items are summarized for a total score, and higher scores reflect more severe auditory hallucinations.

*Beliefs About Voices Questionnaire-Revised (BAVQ-R)* (81). The BAVQ-R is a 35-item self-report questionnaire that measures perceptions about, and emotional and behavioral response to auditory verbal hallucinations. The items are rated on a 4-point scale ranging from 0 (disagree) to 3 (strongly agree). The questionnaire consists of five subscales measuring different meanings given to the voices: *omnipotence* with six items (e.g., “My voice is very powerful”), *malevolence* with six items (e.g., “My voice is persecuting me for no good reason”), *resistance* with nine items (four items for *emotion*: e.g., “My voice frightens me” and five items for *behavior*: e.g., “When I hear my voice usually I tell it to leave me alone”), *benevolence* with six items (e.g., “My voice wants to help me”) and *engagement* with eight items (four for *emotion*: e.g., “My voice makes me feel calm” and four for *behavior*: e.g., “I seek the advice of my voice”).

*Voices Acceptance and Action Scale (VAAS)* (82). The VAAS is a 31-item self-report questionnaire that measures acceptance-based beliefs (defined as a willingness on the part of the voice hearer to have voices in his or her life coupled with an effective, non-avoidant disengagement from them) and action-based beliefs (defined as behaviors that are self-directed rather than being a reaction to the voices). Both the 16 acceptance-based items (e.g., “My voices are just one part of my life”) and the 15 action-based items (e.g., “My voices stop me from doing things I want to do”) are scored on a 5-point scale: *strongly disagree, disagree, unsure or neutral, agree, or strongly disagree*.

*Choice of Outcome in CBT for Psychoses (CHOICE)* (83). This outcome measure was developed to be sufficiently generic to apply across different CBTp approaches and models, but sensitive enough to capture change. It consisted of a two-dimensional 24-item self-report questionnaire, which provides measures for severity and satisfaction across a range of problems/difficulties (e.g., “ways of dealing with distressing experiences [e.g., beliefs, thoughts, and voices],” “the ability to approach problems in a variety of ways”).

### Auditory Paradigm

ERP test sessions occurred in the morning (8–11 a.m.) following overnight abstinence of drugs, alcohol, caffeine and food. During the auditory stimulation, participants sat upright and viewed a silent video (The Blue Planet by BBC, 2001). In the optimal MMN paradigm (84), which was designed to elicit MMN responses to 5 separate auditory deviants, auditory tonal stimuli of 70 dB sound pressure level (SPL) were presented binaurally through headphones and consisted of standard (p=0.5) stimuli (composed of three sinusoidal partials of 500, 1000, 1500 Hz, 75 ms duration) that were randomly intermixed with deviant (p=0.5) stimuli. Stimulus onset asynchrony (SOA) was fixed at 500 ms. The deviant tones differed from the standard tones in terms of pitch, duration, intensity, perceived location of sound origin, or contained a silent gap in the middle of the tone (i.e. gap deviants). The duration deviant was only 25 ms in duration (instead of 75 ms). Half of the pitch deviants were 10% lower (composed of 450, 900, and 1350 Hz partials) and the other half were 10% higher (composed of 550, 110, 1650 Hz partials). Half of the intensity variants were at 80 dB and the other half at 60 dB. A change in perceived location was created by creating an 800 µs time difference between channels, leading to a sensation of a change in location of approximately 90°. Half of the deviants had an 800 µs delay in the right channel while the other half was in the left channel. In the gap deviants 7 ms (including a 1-ms rise and fall) were removed from the middle of the standard stimulus. Stimuli were presented in 3 sequences of 5 minutes each (1845 stimuli) for a total of 15 minutes (5535 stimuli). Each sequence started with a 15 standard tones, followed by a sequence in which every second tine was a standard (p=0.5) and every other tone was one of the five deviants (p=0.1 each). One deviant of each category was presented once every five deviants and deviants of the same category were never presented consecutively.

### ERP Procedures

**Figure 2:**
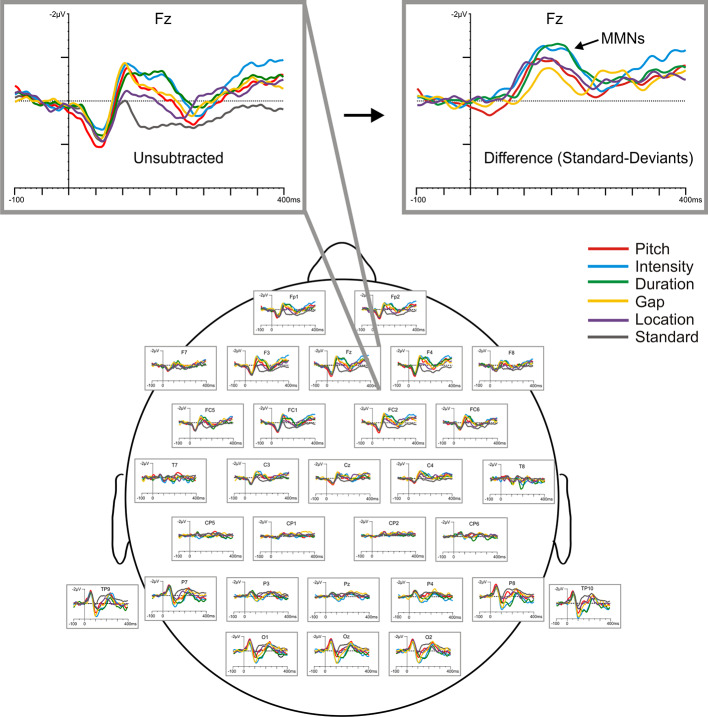
Baseline (raw) grand-averaged ERP waveforms elicited by the standard and five deviant stimuli for all participants (N=24), shown across scalp sites and highlighted at F_z_ with respect to both raw (unsubtracted) and subtracted (deviant minus standard) difference waveforms.

ERPs were recorded with a cap embedded with Ag^+^/Ag^+^Cl^−^ electrodes (EasyCap, Herrching-Brieibrunn, Germany) positioned on 32 (see **Figure 2**) according to the 10–10 system (85). An electrode on the nose served as reference and a ground electrode was positioned at the AF_z_ electrode site. Electrodes were placed above and below the right eye to record vertical electrooculographic (VEOG) activity. Electrical recordings were carried out using a Brain Vision QuickAmp^®^ (Brain Products, GmbH, Munich, Germany) amplifier and Brain Vision Recorder^®^ (Brain Products GmbH, Munich, Germany) software. Electrical activity was sampled at 500 Hz, with amplifier bandpass filters set at 0.1 to 100.0 Hz. Electrode impedances were kept below 5 kΩ.

Off-line analysis was performed with Brain Vision Analyzer^®^ software (Brain Products, GmbH, Munich, Germany). For each stimulus, electrical epochs of 500 ms duration (beginning 100 ms prior to stimulus onset) were digitally filtered (0.1–20 Hz) (86), ocular (87) and baseline corrected (relative to the pre-stimulus segment), and only epochs with EEG voltages ± 75 µV were used for final ERP averages, which were constructed separately for the standard and each deviant stimulus type at each electrode site. Waveforms for the low and high pitch deviants, those for the low- and high-intensity deviants, and those for the right and left location, were averaged together. The mean number of epochs for MMN averages was not significantly different between deviants, nor were there differences in epoch numbers (for each deviant) across test sessions or between treatment groups.

MMNs elicited by frequency (fMMN), duration (dMMN), intensity (iMMN), gap (gMMN), and location (lMMN) deviants were analyzed with difference waveforms, which were derived by digital point-by-point subtraction of the standard stimulus values from those elicited by each of the deviant stimuli. Grand average waveforms, raw and subtracted, are displayed in **Figure 2**. MMN amplitude was defined as the most negative peak (± 5 ms) between 120 and 250 ms at the frontal electrodes (F_3_, F_z_, F_4_), the sites exhibiting maximum MMN amplitudes. Amplitude of the N1 component (peak negativity between 90 and 120 ms) elicited by the standard stimulus was also measured (from F_z_) as an index of sensory registration, which is typically reduced in chronic SZ (88).

### Source Localization

Intracortical current density (A/m^2^) measures at peak MMN activity (based on ERP grand averages) from predefined ROIs was computed using validated (89) exact low-resolution electromagnetic tomography software (eLORETA, version 2081104) (90, 91). eLORETA models the cortical gray matter as a collection of voxels (6239 voxels with a spatial resolution of 5-mm^3^). Relying on the standard electrode positions displayed on the scalp (92, 93), the digitized Talairach atlas (94), the average MRI brain template (MMI152) provided by the Montreal Neurological Institute (95) and a cortically restrained solution space, it calculates within a realistic head model (96) the non-unique “inverse” problem by computing a three dimensional distribution of intracortical source activity (with zero location error) at each voxel based on surface-level electrical signals. The original LORETA method has received considerable validation from studies using EEG (97) and more established localization methods such as structural and functional MRI (98–100) and intracranial electrode recordings (101). Employing the ROI-Extractor tool, the selected ROIs were based on eLORETA-defined Brodmann Areas (BA), and current density data from a single centroid representative voxel of each BA (the voxel closest to the center of the BA mass, which is an excellent representation of the corresponding BA) were extracted for further analysis. This included the pAC (BA 41) and secondary (sAC) auditory cortex (BA 42).

### Statistical Analyses

Statistical analysis was conducted using SPSS version 23 (SPSS Inc., Chicago IL, USA). Two sets of analyses were carried out: 1) the primary set compared data between two groups, including the 13 patients completing the CBTv treatment arm and the 11 patients completing the TAU treatment arm; 2) the secondary set combined data from two groups, including the patients assigned to the CBTv treatment arm and the TAU patients who went on to receive CBTv. For the primary analyses, MMNs were assessed with separate mixed analysis of variance (ANOVA) for each deviant, each ANOVA consisting of one between-group factor with 2 levels (CBTv vs. TAU) and two within-group factors, including time (baseline vs. follow-up) and frontal electrode site (left [F_3_], central [F_z_], and right [F_4_]). MMN latency (at F_z_ only) for each deviant and clinical rating/questionnaire scores were analyzed with similar ANOVAs but with no site factor. Measured as peak negativity in an 80 to 120 ms window, the N100 amplitude/latency values derived from the standard stimulus were also subjected to similar ANOVAs to determine if CBTv affected simple sensory registration. For the deviants exhibiting significant treatment-induced changes in MMN in the between-group analyses, the eLORETA-derived CD values for the pAC and sAC were analyzed using ANOVAs involving a between-group factor and two within-group factors, including time and ROI (BA41, BA42). For the secondary set of analyses, which assessed measures in the combined CBTv treatment group, ANOVAs did not contain a between-group factor. In order to maintain a constant 5 month period between baseline and follow-up sessions in these analyses, the data from the assessments conducted at the initial follow-up session in the TAU group served as their baseline data. For both sets, regardless of whether significant Greenhouse-Geisser corrected (p < 0.05) treatment, time or interaction effects were observed or not, treatment change was assessed *via a priori* planned comparisons of baseline vs. follow-up data. For the deviant MMNs and CDs exhibiting significant treatment effects in this between-group analysis, Spearman's rho correlation coefficient statistic was used to examine the relationship between changes in electrophysiological measures and changes in clinical/questionnaire measures, as well as between MMN changes and source localized CD changes. In order to reduce the number of statistical tests, these correlations were assessed only for electrophysiological and clinical/AVH measures showing CBTv treatment effects in the initial set of analyses.

## Results

Of the fourteen patients assigned to CBTv, thirteen (8 males) completed all assessments at baseline and follow-up and provided usable EEG recordings. Of the eleven patients assigned to TAU, ten (6 males) completed all assessments at baseline and follow-up and provided usable EEG recordings. Thus, the attrition rate was 8%, with onset of medical illness (one patient) and unusable EEG data (one patient) accounting for patient-drops. During their subsequent participation in CBTv, only six of the ten patients in the TAU group completed all assessments at baseline (i.e., some data as from their follow-up session post TAU) and at follow-up and provided usable EEG recordings. Patent-drops were due to either change in medication (one patient), or unusable EEG (two patients). The final CBTv and TAU groups were similar in age, gender, year of education, duration of illness, PANSS positive, PANSS negative, PANNS total and PSYRATS total scores (**Table 1**).

**Table 1:** Demographic and clinical measures for treatment groups.

Demographics	CBTv group (n = 13; 7 males)		TAU group (n = 10; 6 males)	
	Mean ± SD Baseline	Mean ± SD Follow-up	Mean ± SD Baseline	Mean ± SD Follow-up
Age (years)	41.77 ± 14.69		47.8 ± 11.81	
Education (years)	4.62 ± 1.33		5.5 ± 1.18	
Duration of illness (years)	16.1 ± 11.64		21.78 ± 9.60	
PSYRATS total	25.15 ± 5.38	22.53 ± 6.21	27.5 ± 4.62	27.89 ± 4.76
PANSS				
Positive Scale	15.62 ± 3.31	16.2 ± 4.41	15.6 ± 3.53	16.17 ± 5.94
Negative Scale	15.77 ± 4.78	16.2 ± 4.59	15.5 ± 4.17	13.7 ± 5.28
General	33.31 ± 13.71	30.3 ± 6.17	31.3 ± 6.43	31.67 ± 7.71

PSYRATS, The Auditory Hallucinations subscale from the Psychotic Symptom Rating Scale; PANSS, Positive and Negative Syndrome Scale.

Follow-up for the PANSS measures included missing data (3 missing cases from the CBTv group and 4 missing cases from the TAU only group).

### CBTv Effects on Clinical/AVH Symptoms

#### Between-Group Analyses

The CBTv group did not differ from the TAU group with respect to changes in PANSS positive, negative or general symptoms (**Table 1**). Similarly, no group differences were observed with respect to changes in the frequency and quality of AVH symptoms assessed with PSYRATS ratings (**Table 1**).

**Figure 3:**
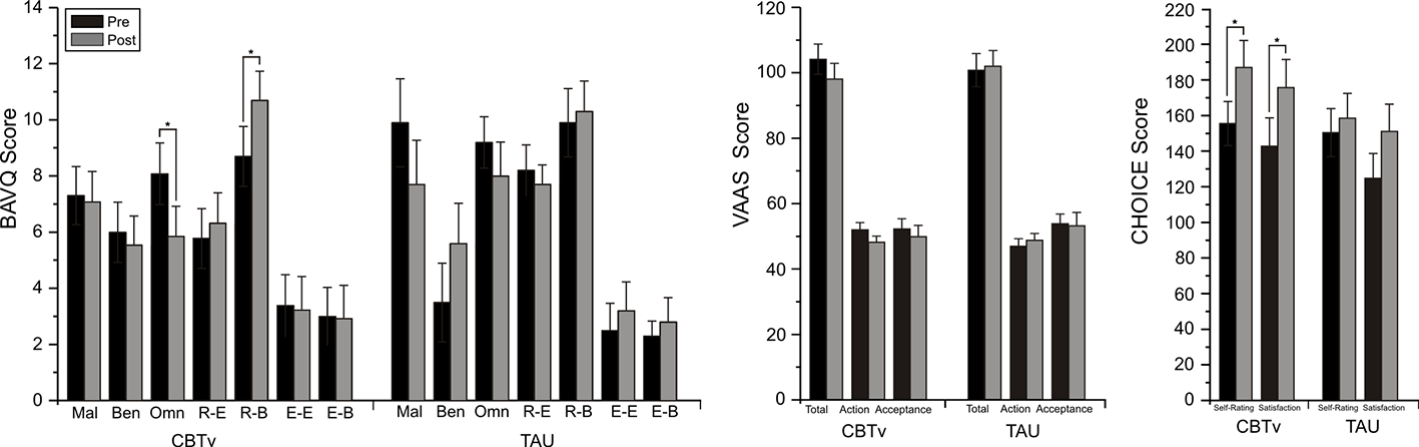
Mean (± SE) rating scores for BAV-Q, VAAS, and CHOICE instruments administered to patients in CBTv and TAU conditions at pre- (baseline) and post-treatment.

For the BAVQ-R, significant time effects were observed for two of the subscale scores, omnipotence (F = 7.36, df = 1/21, p = 0.013) and resistance behavior (F = 4.37, df = 1/21, p = 0.049). Planned comparisons found these reductions in omnipotence (p = 0.014) and increases in resistance behavior (p = 0.015) ratings between baseline and follow-up to be limited to the CBTv group (**Figure 3**). Analysis of VAAS rating failed to yield any significant group, time or interaction effects but ratings scores on both the CHOICE severity (F = 8.08, df = 1/21, p = 0.01) and CHOICE satisfaction (F = 12.16, df = 1/21, p = 0.002) dimensions showed significant time effects, with planned comparisons showing significant changes in severity (p = 0.003) and satisfaction (p = 0.008) only in the CBTv group (**Figure 3**).

#### Combined Group Analyses

**Figure 4:**
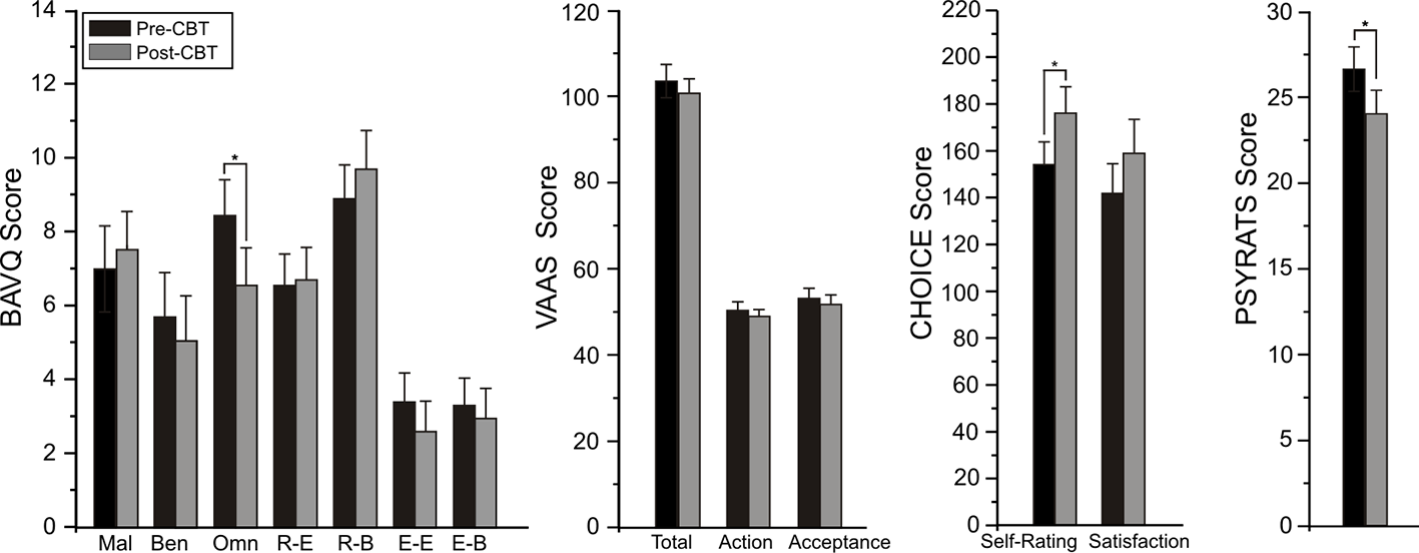
Mean (± SE) pre- (baseline) and post-treatment rating scores for BAV-Q, VAAS, and CHOICE instruments administered to all patients (N=22) completing CBTv.

Changes from pre- to post-CBTv were not observed for PANSS or VAAS ratings but significant reductions were shown for total PSYRATS (*p*=0.035), BAVQ-R omnipotence (*p*=0.0013) and CHOICE severity (*p*=0.009) rating scores (**Figure 4**).

### CBTv Effects on MMN/N100 Amplitude/Latency

#### Between-Group Analyses

**Figure 5:**
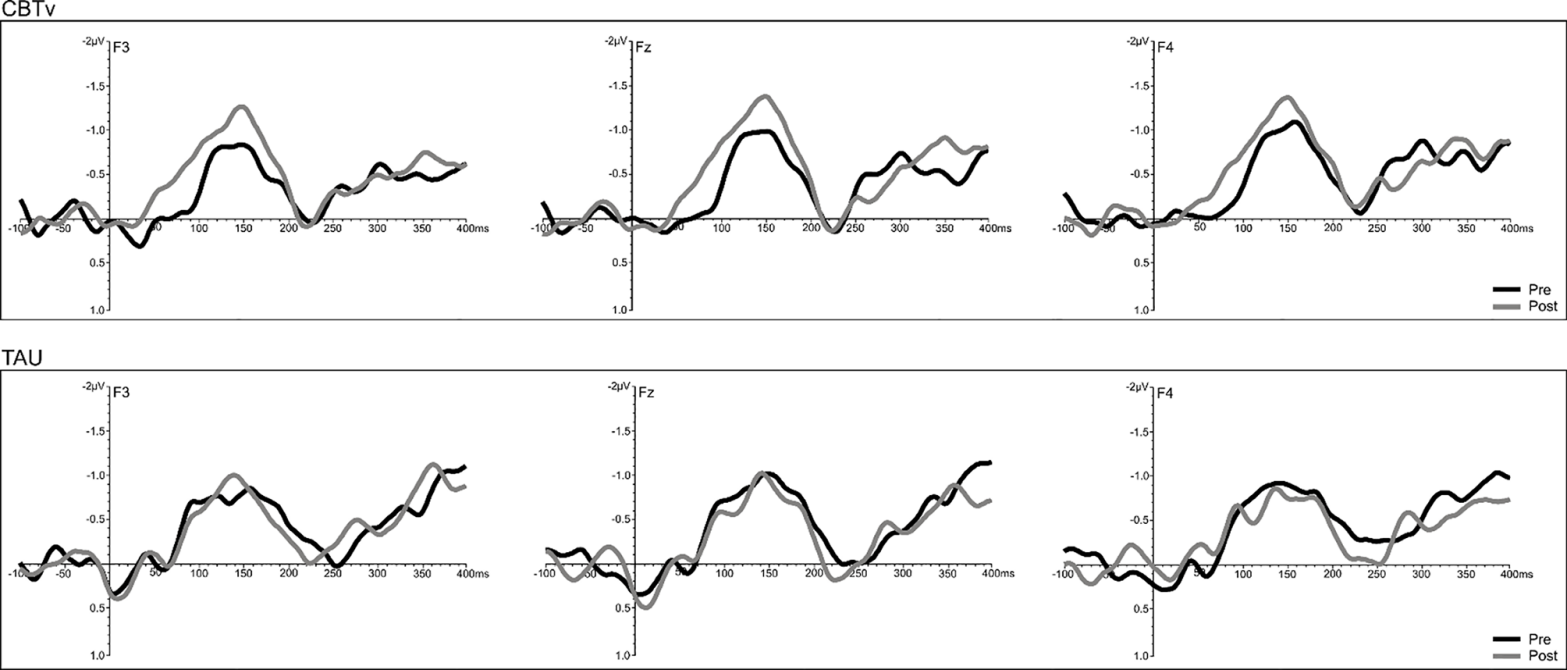
Grand-averaged subtracted pitch deviant waveforms at frontal (F_3_, Fz, F_4_) sites recorded pre- (baseline) and post-treatment in patients assigned to CBTv and TAU conditions.

**Figure 6:**
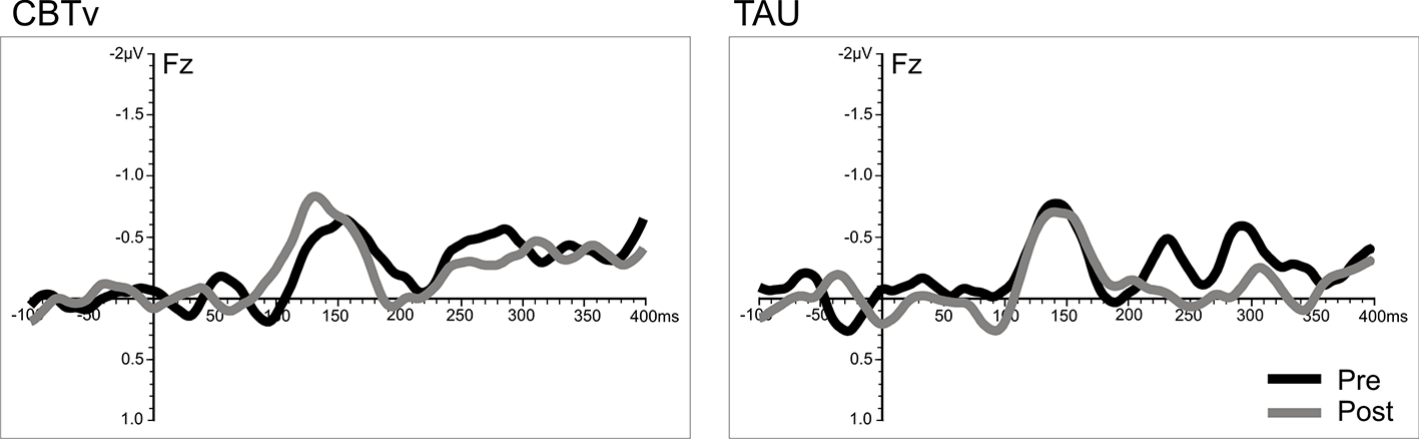
Grand-averaged subtracted gap deviant waveforms at mid-frontal site (Fz) recorded pre- (baseline) and post-treatment in patients assigned to CBTv and TAU conditions.

Analysis of frontal amplitudes did not reveal any significant group, treatment, or time effects for MMN elicited by intensity, duration, gap or location deviants. A significant treatment x time interaction (F = 15.78, df = 1/40, p = 0.001) was shown for the pitch deviant, with planned comparisons revealing significant increases in pMMN amplitudes in the CBTv group at follow-up compared to baseline (p = 0.001) as well as greater pMMN amplitudes in CBTv group compared to TAU group p = 0.043) at follow-up (**Figure 5**). Analysis of MMN latency yielded a significant treatment x time interaction for the gap deviant (F = 4.41, df = 1/25, p = 0.049) with planned comparisons showing a reduced (earlier) gMMN latency (**Figure 6**) in the CBTv group (p = 0.019) at follow-up (M = 148.68 ms, SE ± 6.86) compared to baseline (M = 164.02 ms, SE ± 6.92). Neither the amplitude nor latency of N100 were affected by treatment.

#### Combined Group Analyses

**Figure 7:**
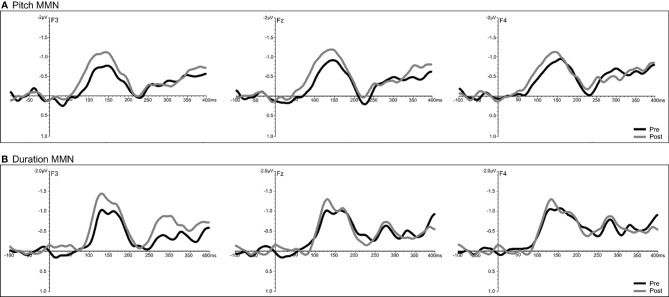
Grand-averaged subtracted pitch **(A)** and duration **(B)** deviant waveforms at frontal (F_3_, F_z_, F_4_) sites recorded pre- (baseline) and post-treatment in all patients (N=22) completing CBTv.

A significant time effect was observed only for the pitch deviant (F = 14.68, df = 1/18, p = 0.001), with pMMN amplitudes showing an increase at follow-up compared to baseline (**Figure 7**). Analyses of the duration deviant yielded a significant time x electrode interaction (F = 9.12, df = 1/36, p = 0.002), with comparisons of left frontal (F3) amplitude showing a greater dMMN amplitude (p = 0.029) at follow-up compared to baseline (**Figure 7**). For MMN latency, analysis showed a significant time effect for the duration deviant (F = 6.71, df = 1/18, p = 0.018), with dMMN exhibiting a shorter latency (M = 153.01 ms, SE ± 7.11) at follow-up compared to baseline (M = 166.46 ms, SE ± 6.03) latency (**Figure 7**). No treatment effects were observed for N100 amplitude or latency.

### CBTv Effects on Source Localized CD

#### Between-Group Analyses

**Figure 8:**
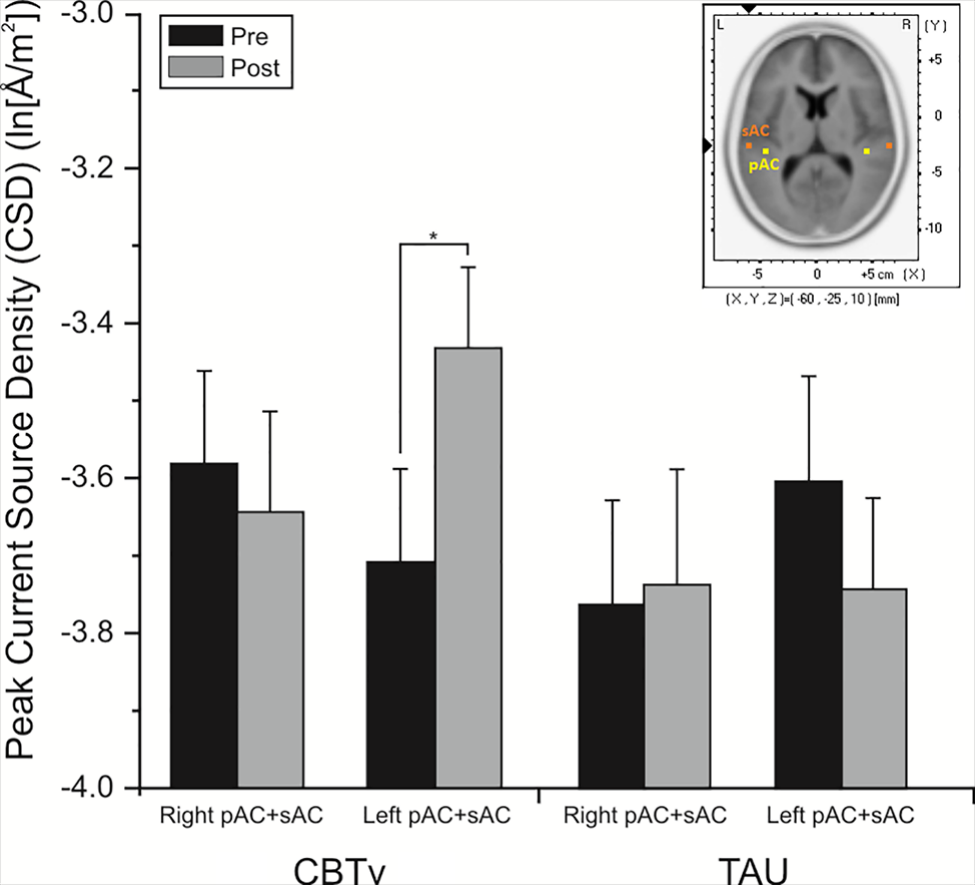
Mean (± SE) current density values (ln[Å/m^2^]) of the left and right hemisphere of the auditory cortex (pAC and sAC combined) in patients assigned to CBTv and TAU conditions.

For the pMMN, analysis of localized CD yielded a significant region effect (F = 33.53, df = 1/21, p = 0.001), with CD of the sAC being greater than CD of the pAC. A significant treatment x time x hemisphere interaction was also evidenced (F = 11.62, df = 1/21, p = 0.008), with planned comparisons finding increases (p = 0.008) in CD in the left hemisphere of the CBTv group at follow-up compared to baseline (**Figure 8**).

#### Combined Group Analyses

**Figure 9:**
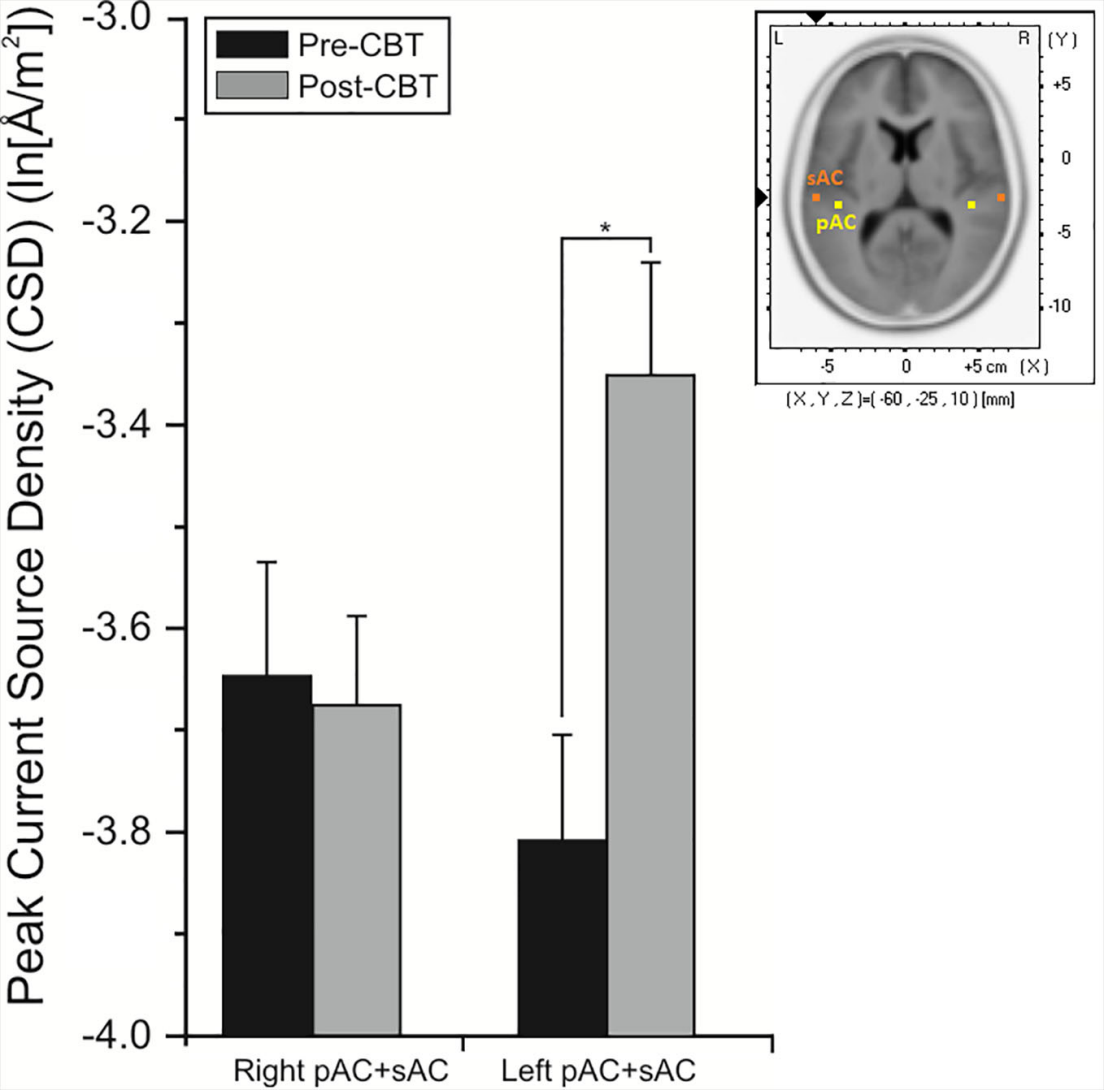
Mean (± SE) current density values (ln[Å/m^2^]) of the left and right hemisphere of the primary and secondary auditory cortex in all patients (N=22) completing CBTv.

Analysis of CD associated with the pMMN showed significant (F = 17.85, df = 1/18, p = 0.001) region effect, with CD in the sAC being greater than CD in the pAC. In significant time (F = 5.38, df = 1/18, p = 0.032) and time x hemisphere interaction effects (F = 8.27, df = 1/18, p = 0.010), planned comparisons showed significant overall increases (p = 0.002) in CD of the left auditory cortex in the CBTv group at follow-up compared to baseline (**Figure 9**).

### Relationships Between Symptoms and MMN/CD

**Figure 10:**
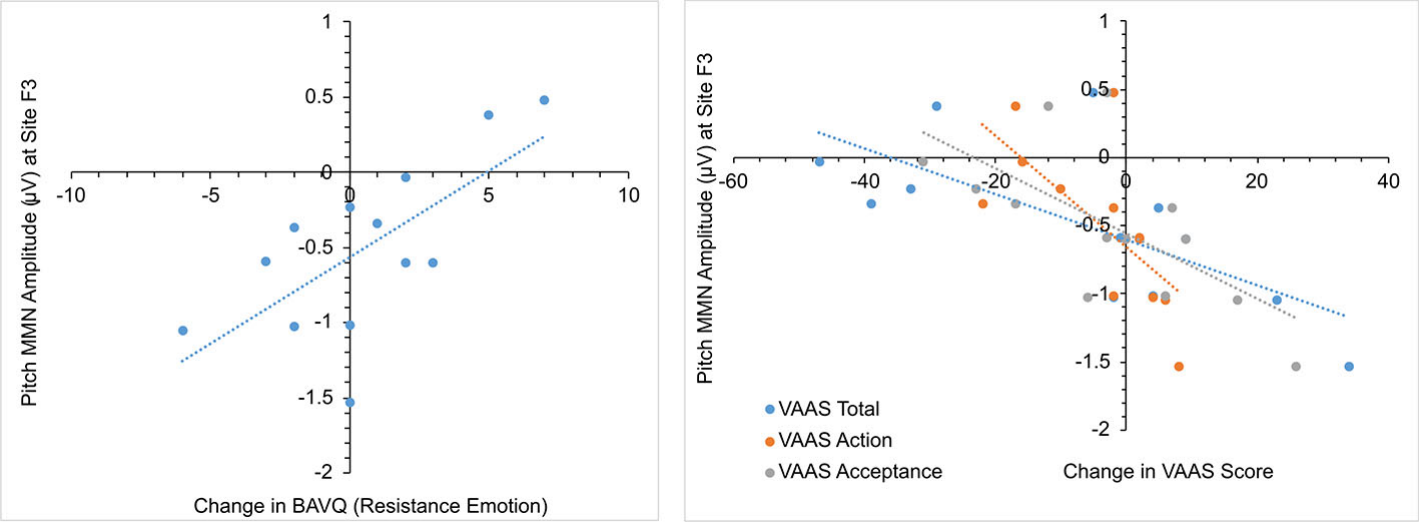
Scatterplots showing relationships between pitch MMN amplitude change and BAV-Q and VAAS ratings change in the initial group receiving CBTv.

**Figure 11:**
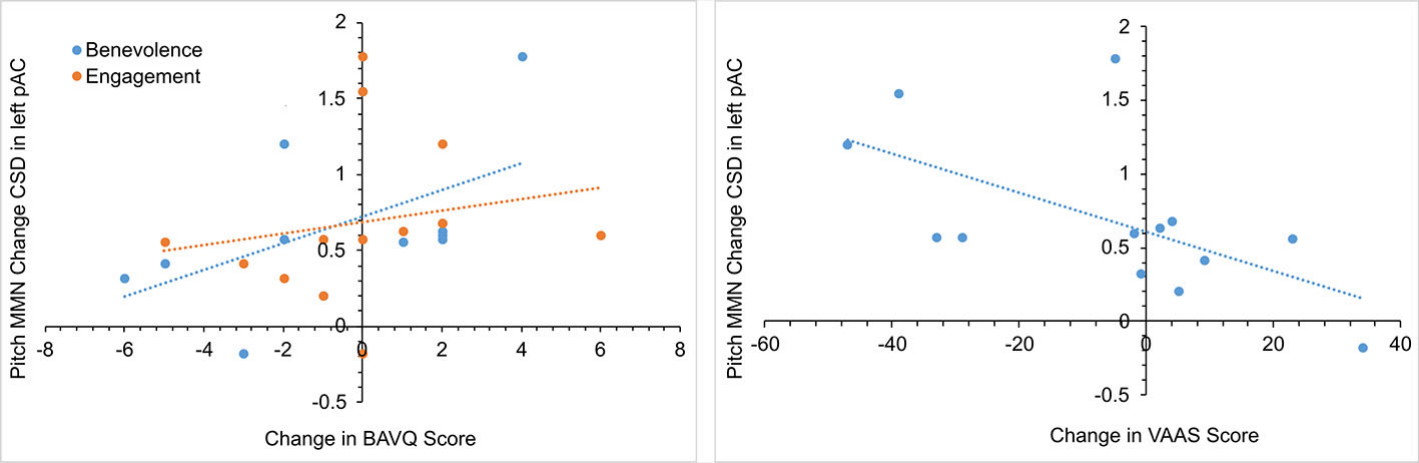
Scatterplots showing the relationships between changes in pitch MMN current density (CSD) in the left primary auditory cortex (pAC) and BAVQ-R and VAAS rating changes in the initial group receiving CBTv.

In the initial CBTv group, changes in the pMMN amplitude (from baseline to follow-up) were positively correlated with changes in the resistance emotion subscale of the BAVQ-R (r = 0.64, p = 0.029), and negatively correlated with the total (r = −0.76, p = 0.002) and both the activation (r = −0.82, p = 0.001) and acceptance (r = −0.70, p = 0.008) scores of the VAAS (**Figure 10**). Treatment-induced changes in symptoms were found to be related to treatment-induced changes in CD of the auditory cortex but only in the left pAC. In this region of the auditory cortex, CD changes were positively correlated with changes in the benevolence (r = 0.63, p = 0.021), resistance emotion (r = 0.55, p = 0.05) and engagement behavior subscale scores (r = 0.54, p = 0.04) of the BAVQ-R, and negatively correlated with changes of the total VASS(r = −0.68, p = 0.01) score (**Figure 11**).

### Relationships Between MMN and CD

In the initial CBTv group, correlations between the treatment changes in the pMMN and the associated CD were limited to the left hemisphere, with increases in fMMN at F3 being positively correlated with increases in CD in the left pAC (r = 0.58, p = 0.01), left sAC (r = 0.53, p = 0.02) and in combined left auditory cortical regions (r = 0.55, p = 0.01).

## Discussion

This pilot study shows changes in EAIP during a pure tone auditory oddball paradigm in a sample of patients with SZ and persistent AVHs, attributable to the effects of CBTv. Compared to patients receiving TAU in the initial analyses, the patients who underwent CBTv showed significantly greater increases in auditory deviance detection as evidenced in enhanced MMN response to pitch deviants and faster (earlier latency) MMN responses to auditory gap deviants at treatment follow-up. These changes occurred independently of general psychiatric symptoms (PANSS) and changes in sensory registration (N100). The patients that received therapy also showed significant increases in activation (CD) of the left auditory cortex during the processing of auditory pitch deviants. Although CBTv did not affect self-reports of the frequency or quality of AVHs (PYSRATS), or the severity of psychotic symptoms (PANSS), it improved patients' perceptions and behavioral response to AVHs (BAVQ-R), and CBTv was thought by patients to be associated with better outcome (CHOICE) than TAU. Even more interestingly, these clinical changes together with self-reported improvements in patient beliefs about AVHs (VAAS) at CBTv follow-up were associated with increases in MMN response and left auditory cortex activation to pitch deviants.

Targeting the cognitive appraisals, perceptions, and beliefs concerning the nature of psychotic symptoms, both individual CBT (41) and group CBT in SZ (102) have been effective in alleviating positive psychotic symptoms as a whole (i.e. hallucinations and delusions) but, as with our own negative findings, the efficacy of CBT interventions specific for hallucinations has been mixed in regards to reductions in the frequency and severity of these symptoms (103, 104). Similar mixed findings in SZ have been reported in CBT trials with added ACC and ATT techniques (105). Our augmented CBTv did however affect AVH-related beliefs and behavioral reactions to hallucinations, shown by reductions in BAVQ-R Omnipotence and BAVQ-R Resistance Behavior scores, which may in part have accounted for patients' favorable outcome perception (CHOICE) with this therapy.

Reductions in conviction of beliefs about the power/authority (omnipotence) of voices and compliance to voices have been observed in previous group CBTp trials (105–107) and are clinically important considering that our patients' hallucinations have failed to respond to effective antipsychotic drugs. Employing specialized interventions like CBTv to target beliefs about voice omnipotence, particularly with respect to commanding aspects of the voices, which have been linked to a range of dangerous behaviors (aggression, violence, self-harm, and suicide), is a therapeutically relevant goal as voice omnipotence predicts compliance to hallucinations, and reductions in these beliefs about voices are associated with reduced cognitive functioning given their negative relationship with exogenous attentional processes (26, 108–110).

Consistent with our hypotheses that dampening the impact of internal voice processing with CBTv would result in the enhanced sensory processing of external auditory stimuli, improvements in EAIP with therapy were evidenced by larger pMMN and shorter gMMN responses in SZ patients with persistent AVHs. Rarely examined with other deviant types, abnormal deviance detection in SZ has been most frequently documented with pitch and duration, and occasionally with intensity changes in simple sound stimuli (17, 111, 112). While pMMN is thought to be sensitive to illness duration/disease progression, the dMMN behaves more as a trait index and as a valid endophenotype reflecting greater vulnerability to illness (17). Although not consistently observed, attenuated pMMN, dMMN, and iMMN have been observed in our laboratory in hallucinating (vs. non-hallucinating) SZ patients and have been correlated with hallucinatory severity ratings (19–22, 113, 114).

Possibly reflecting contributions from bilateral prefrontal cortices, the auditory MMN response to both simple sound and speech deviants is mainly dependent on synaptic plasticity mediated by glutamatergic *N*-methyl-d-aspartate (NMDA) receptors in the auditory cortex (115–117). Although also shown with the detection of duration deviants in our combined group analysis, the more reliable improvements in deviance detection with CBTv were observed in response to the pitch deviant, with pMMN being shown to be increased in both sets of analyses along with pitch CD in auditory cortices. Not always associated with auditory hallucinatory experiences, SZ is associated with deficits in the perception of a broad range of auditory features, including pitch discrimination as measured in tone-matching tasks (118). Dependent on the low-level acoustic features or type of complex naturalistic sound, the neural representation and processing of acoustic stimuli is confined to different regions within the human auditory system. The neural mechanisms underlying pitch perception are still largely debated but are assumed to involve a hierarchy of pitch processing steps, starting in the subcortical structures and terminating at the cortical level, where perceived pitch (variations) is most likely encoded (119). Certain areas of the auditory cortex are specifically sensitive to pitch, and although the locations are still another debate (120), previous functional neuroimaging has identified pitch-coding regions, including anterior-lateral pAC (on Heschl's gyrus [HG]) and adjacent sAC processing areas (on the superior temporal gyrus [STG]) (121), and extending to the planum temporale during the passive influences of infrequent pitch changes (122, 123). Increased pMMN (and dMMN) amplitudes with CBTv in patients experiencing persistent AVHs is consistent with brain volumetric studies showing negative relationships between left hemisphere HG/STG volume and both hallucination severity (122, 123) and MMN amplitude in SZ patients (124).

Changes in the appraisal of and response to AVHs with CBTv were associated with enhanced pMMN amplitudes and selective increases in pitch CD in the left pAC, and are consistent with the current status of AVHs in that they implicate speech perception areas in the left temporal lobe, improving perception of and attention to external sounds. These relationships between changes in auditory neural responsiveness and AVH symptom ratings are in line not only with structural studies showing the left pAC gray matter volume reduction in SZ to be associated with AVH severity (122, 123, 125), but also with functional neuroimaging studies confirming a “paradoxical” brain activation in relation to AVHs—the left pAC evidencing increased activation (during silent rest) in the absence of an external stimulus, and decreased activation in the presence of an external auditory stimulus (11). Although the specific brain mechanisms underlying these alterations are not understood, it is reasonable to speculate that they may be mediated by changes in glutamate neurotransmission. Aberrant glutamate levels in temporal and prefrontal cortical areas are found in SZ with frequent and severe hallucinations (126, 127) and glutamate receptor mediated synaptic plasticity in the pAC, as indexed by MMN alteration, is compromised in patients with SZ and particularly in patients with AVHs. Although not necessarily affecting tonic glutamate levels, changes in MMN responsiveness to pitch deviance with CBTv and adjunctive ATT and ACC techniques may indicate an increased ability to adequately modify synaptic plasticity in response to auditory (glutamate) neurotransmission resulting from external auditory stimulation.

The co-occurrence of altered ratings of beliefs/responses to AVHs together with changes in neural response (pMMN) to auditory deviance, which was shown to be statistically correlated with changes in beliefs, behavioral/emotional (BAVQ-R) and coping response (VAAS) to hallucinations, may be tentatively explained by the influence of CBTv on a common underlying NMDA receptor-mediated process—prediction error signaling. In a predictive coding framework, predictive coding is viewed as a hierarchical information processing model which posits interactions between lower-order (bottom-up) perceptual signals and higher-order cognitive processes in a dynamic, interactive fashion to generate predictions about the environment and compare incoming stimuli with these predictions (128, 129). Within this model, neural responses to stimuli that match predictions are suppressed, while stimuli that are unexpected, violating these predictions, trigger a mismatch “prediction error” signal, which signals that updating of expectations is required to accommodate the discrepant stimuli (130–132). It has been proposed that in hallucinating patients, excess aberrant spontaneous activation of the auditory sensory cortex may be confused by the brain with activity typically seen with external auditory stimulation, leading to erroneous expectations of a perceptual event (predictive coding failure) with the brain inferring externally located voices which in turn leads to a false (AVH) perception (129, 133, 134). Predictive tone signaling has been observed at the earliest levels of auditory cortical hierarchy—in the pAC (135). The MMN is hypothesized to reflect a prediction error signal (e.g. the properties of the deviant stimulus do not match the predictive model formed by the train of preceding standards, thus the model must be updated in order to improve predictive accuracy) (136), which is attenuated during NMDA receptor antagonist treatment with ketamine (137–139) and can be used to examine abnormalities in predictive coding. In a roving standard MMN paradigm, which allows for optimal evaluation of prediction errors (140), MMN deficits in SZ have reflected attenuated prediction error signaling (141). This is particularly pronounced in hallucinating (v. non-hallucinating) patients and consistent with a predictive coding account of hallucinations in SZ (142).

Hallucinatory experiences are associated with hyper-activation of the primary and secondary sensory areas, possibly due to dysregulation related to frontal lobe hypo-activation. Different brain mechanisms appear to underlie the clinical effects of pharmacotherapy and psychotherapy (143, 144). It has been argued that psychotherapeutic approaches such as CBT may exert their affects by gaining control of the function of particular circuits, such as changes in appraisal, control of attention, modulation of interceptive processes, and may involve key nodes, such as anterior cingulate and medial prefrontal areas (involved in error detection and conflict monitoring), dorsolateral prefrontal cortex areas (involved in cognitive control/working memory), and insula (interceptive sensitivity) (105). We can also speculate that different psychotherapeutical strategies may have different brain effects within a circuit. In our augmented treatment protocol, CBT and AAC focused on controlling the emotional response to hallucinations might be based on decreasing endogenous brain activity in the temporal/limbic areas, while ATT focused on auditory perceptual processes might be based on increasing externally induced brain activity in these same regions. Although not directly comparable with ATT, targeted cognitive training (TCT) of the auditory system in SZ patients has been shown to drive plasticity in cortical activation patterns related to both sensory representations as well as higher order cognitive processes (145, 146). In patients with SZ, TCT has not only produced significant improvements in auditory perception and learning, which was predicted by MMN (147), but also increases in verbal learning and reductions in AVHs (148, 149). Improvements in higher order auditory processing gained through TCT in SZ are dependent on the severity of basic auditory deficits (150). Given that the MMN response to deviant sounds has also been shown to have a direct mediating effect on cognition and functional outcome in SZ patients (151), future research may want to examine the effects of pairing CBTv with TCT aimed at improving auditory discrimination as a potential optimal strategy that would benefit both AVH symptoms and cognitive and psychosocial functioning.

## Limitations

This study has several limitations. Regarding adherence to CBTv, a fidelity/treatment response scale was not used, and future research would benefit from audiotaping (with consent) and using a fidelity measure and independently trained raters. Although the results are relatively consistent across the two sets of analyses, they require replication in a larger sample. Patients were randomized to CBTv and TAU vs TAU only conditions but blindness was not a component of the study design and should be an aim in future work. Concurrent antipsychotic treatment may have influenced the results, but both groups of patients were receiving similar treatments. In order to reduce Type 1 error rates our source analysis with eLORETA was limited to two ROI, and additional studies are required to examine CBTv effects on non-auditory brain regions, especially frontal areas which are thought to contribute to MMN generation during deviance detection and to interact with auditory cortices in producing AVHs. External auditory stimulation for MMN generation was limited to pure tone deviants and additional studies need to examine CBTv effects on the processing of complex natural sounds including MMN response to speech deviants, which appear to be particularly sensitive to EAIP dysfunction in patients with SZ (21, 152) and as with MMN to response to pure tone deviants are reflective of NMDA receptor-mediated neurotransmission in auditory cortices (117). Functional neuroimaging has shown that the neural encoding of natural sounds (e.g., speech, voice) entails the formation of multiple representations of sound spectrograms with different degrees of spectral and temporal resolution (152–154). Combining the superior temporal and spatial resolution of EEG and fMRI techniques, respectively, to image neural activity during resting-state (absence of external auditory stimulation) and in response to behaviorally relevant, real-world sound stimuli would be an optimal strategy for achieving a more complete picture of brain mechanisms involved in AVH responsiveness to CBTv in SZ patients (155–160).

Finally, we assessed responsivity to external auditory stimulation on the neural level and there is a need to incorporate behavioral assessments (e.g., tone-matching and dichotic listening tasks) in order to examine performance changes associated with auditory processing. Optimally, these would be complemented with tests assessing changes in cognitive and functional outcome with CBTv.

## Conclusion

In conclusion, we have shown for the first time significant changes in MMN responsiveness to external auditory deviants in SZ patients undergoing cognitive therapy for persistent auditory hallucinations. Correlated with improvements in patient's response to hallucinations, these neural findings improve our understanding of how psychotherapy may benefit patients with AVH, possibly by shifts in perceptual processing from internal distressing auditory (voices) stimulation to potentially relevant changes in external auditory stimuli. As these present observations were captured in a relatively small test sample, confirmation of these observations in larger studies integrating biomarkers like MMN to elucidate treatment effect mechanisms will help to increase our understanding of and ability to personalize psychotherapeutic approaches such as CBTv (153, 154).

## Data Availability Statement

The datasets generated for this study are available on request to the corresponding author.

## Ethics Statement

The studies involving human participants were reviewed and approved by Dominique Bourget, Acting Chair, University of Ottawa Institute of Mental Health Research REB. The patients/participants provided their written informed consent to participate in this study.

## Author Contributions

VK and NW were responsible for the conceptualization of the study, NW lead the CBT for patients and was assisted by DS. Recruitment was the responsibility of AL and coordination, testing, and data analysis were performed by HB, DS, SS, and AB. Manuscript was prepared by VK and figures and tables were prepared by SS.

## Funding

This work was supported by a grant from the University of Ottawa Medical Research Fund to NW, VK, and AL.

## Conflict of Interest

The authors declare that the research was conducted in the absence of any commercial or financial relationships that could be construed as a potential conflict of interest.
